# Report of Deaths in the City of Buffalo for the Month of August, 1861

**Published:** 1861-11

**Authors:** J. Whitaker

**Affiliations:** Health Physician


*Report of Deaths in the City of Buffalo for the month of August*, 1861.

Accident 8, Apoplexy 1, Asthma 1, Cancer 1, Cholera Infantum 8, Cholera Morbus 5, Cholera 1, Consumption 17, Convulsions 16, Debility 5, Delirium Tremens 1, Dentition 1, Diarrhœa 9, Disease of the Brain 3, Disease of the Heart 3, Disease of the Lungs 1, Disease of the Spine 1, Dropsy 3, Dropsy of the Brain 5, Dysentery 6, Erysipelas 2, Scarlet Fever 11, Typhoid 5, Gangrene 2, Hæmorrhage 2, Inflammation of the Brain 6, Inflammation of the Liver 1, Inflammation of the Lungs 6, Inflammation of the Lungs Typhoid 1, Inflammation of the Peritoneum 1, Inflammation of the Womb 1, Lead Colic 1, Marasmus 6, Old Age 3, Paralysis 1, Premature Birth 1, Whooping Cough 18, Softening of the Brain 1, Still Born 2, Unknown 8. Total 213. Under 1 year of age 88. Between one year and twenty 73. Between twenty and fifty 35. Over fifty 17. Ages unknown 3. Males 106. Females 103. Not given 4. Total 213. J. WHITAKER, Health Physician.

*Reports of Death in the City of Buffalo, for the month of Sept.* 1861.

J. WHITAKER, Health Physician.

